# Innovative Aesthetic and Functional Rehabilitation in a Two-and-a-Half-Year-Old Using a Modified Groper’s Appliance

**DOI:** 10.7759/cureus.64824

**Published:** 2024-07-18

**Authors:** Nikita Saini, Debapriya Pradhan, Saurabh Tiwari, Raksha Thakur, Namrata Jain

**Affiliations:** 1 Department of Pedodontics and Preventive Dentistry, Hitkarini Dental College and Hospital, Jabalpur, IND

**Keywords:** dental procedures, paediatric dentistry, modified groper’s appliance, primary teeth, functional rehabilitation, aesthetic rehabilitation, early childhood caries

## Abstract

The repercussions of the early loss of primary teeth, be it from trauma or caries, encompass compromised chewing efficiency, diminished aesthetics, potential development of abnormal oral habits, and difficulty in articulation of labiodental sounds, significantly influencing the child's psychological well-being and behavior. Moreover, the untimely loss of posterior teeth results in the loss of space, potentially leading to malocclusion and functional disruptions. Hence, addressing dental rehabilitation in these cases is both a challenge and a necessity. A toddler aged two and a half was brought to our department with a primary concern of multiple caries. Severe decay with 54, 61, 62, and 64 and missing 51 and 52 were seen. The child experienced pain from decayed back teeth, and the parents were distressed about compromised aesthetics caused by decayed front teeth. Given the child's young age and the sufficient time for permanent teeth to emerge, their concerns were heightened. To address these issues, a modified Groper's appliance was crafted to restore aesthetics and functionality. A supplementary crib-like wire component was incorporated into the appliance to facilitate a simpler and aesthetically pleasing composite crown buildup. Satisfaction with the treatment was evident from both the parents and the child. Subsequent follow-up sessions revealed no adverse effects attributable to the appliance, thus concluding that the additional wire component seamlessly integrated into the appliance offers several advantages, including enhanced aesthetics through composite shade matching for a simplified crown buildup. Particularly in remote areas, where obtaining a set of deciduous acrylic teeth can be challenging, this feature proves to be advantageous. It eliminates the necessity to trim permanent acrylic teeth to achieve a deciduous appearance. Additionally, the time and cost associated with the laboratory fabrication of heat-cure acrylic crowns can be circumvented. This distinctive case report underscores the creation of a modified Groper's appliance for the aesthetic and functional rehabilitation of a child grappling with early childhood caries (ECC). The appliance was well received, and no adverse outcomes were observed.

## Introduction

Early childhood caries (ECC) continues to be a prevalent issue affecting children worldwide, influencing not just their oral health but also their overall well-being. It is important to understand that this condition does not exclusively impact children; parents, as responsible caregivers, also feel its effects [1]. ECC is a culmination of the interplay among various risk factors. These include the transmission of streptococcus from mother to child during early stages, the educational background of the parents, nocturnal bottle-feeding, sugar intake between meals, insufficient toothbrushing practices, avoidance of fluoride toothpaste, socioeconomic status, and several other contributing factors [2].

ECC is linked to various health issues, encompassing localized pain, infections, and abscesses, resulting in challenges such as difficulty in chewing, malnutrition, gastrointestinal disorders, and disrupted sleep patterns [3]. Anterior tooth loss is linked to several factors, such as the tilting of neighboring teeth, midline shift, impaired chewing function, speech difficulties, abnormal oral habits, and excessive eruption of opposing teeth [4]. Moreover, in children, it has a significant impact on their psychological well-being. Various aesthetic options, such as removable or fixed partial dentures, offer solutions in this context. Care must be taken to choose a replacement method that does not impede the natural eruption process of the underlying successor teeth [5].

Addressing this, it becomes essential to focus on managing prosthetics to restore all functions, including the child's appearance.

## Case presentation

A two-and-a-half-year-old male child reported to the Department of Paediatric and Preventive Dentistry with the chief complaint of missing and severely decayed upper front teeth and pain in the upper back right and left teeth region since one week prior. The parents shared that the child encountered difficulties such as disrupted sleep and challenges in chewing and biting because of pain, resulting in discomfort and a reduced appetite. This, coupled with weight loss and notable body weakness, heightened their concerns regarding the child's overall health and well-being. Additionally, the parents expressed concern regarding the child’s impaired aesthetics.

The patient's parents provided a dental history, indicating that the extraction of 51 and 52 and pulpectomy of 61 and 62 occurred at a private dental clinic three months prior. This procedure was necessitated because of severe decay and pain. The patient's dietary background indicated a pattern of nighttime bottle-feeding for about a year and a half. During the intraoral clinical examination, decay was observed in multiple teeth in both the maxillary and mandibular arches. An edentulous area corresponding to 51 and 52 was observed.

**Figure 1:**
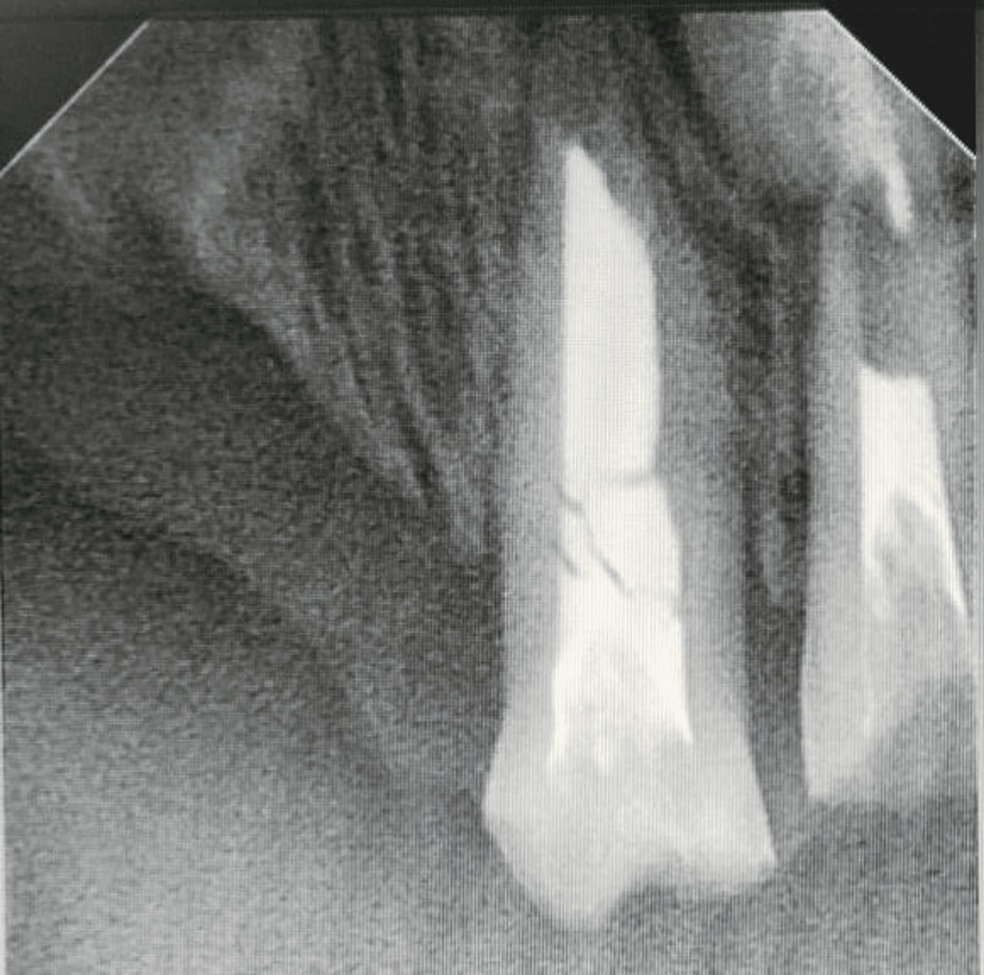
Preoperative radiograph showing missing 51 and 52 and endodontically treated 61 and 62 without any final restoration

To further investigate, intraoral radiographs (radiovisiography) were recommended, revealing carious teeth with pulpal involvement in 54 and 64. Teeth 61 and 62 were endodontically treated without any final restoration. Their roots displayed no extreme resorption and were deemed salvageable (Figure 1).

Initial occlusal caries were also noted in 74 and 84, leading to a diagnosis of severe ECC (S-ECC). The child exhibited negative behavior and reluctance to dental treatment (Frankl Behavior Rating II), stemming from the previous traumatic experience.

Clinical procedure

To ease the patient, a noninvasive treatment approach was implemented during the first dental visit. These procedures included intraoral clinical and radiographic examinations, along with oral prophylaxis for both the maxillary and mandibular arches. Medications, encompassing antibiotics and pain relievers, were prescribed for the patient. The child was scheduled for a follow-up the next day for further treatment. The treatment plan was outlined in a specific order as mentioned below, thoroughly explained to the parents, and written consent was obtained from each.

1) Pulpectomy with 54 and 64, prioritized because of challenges while eating.

2) Re-pulpectomy with 61 and 62, followed by post and direct composite crown.

3) Glass Ionomer cement restorations with 74 and 84.

4) Fluoride application.

5) Adaptation of stainless steel crowns with 54 and 64, succeeded by an impression to create the modified Groper's appliance. Subsequently, the appliance is to be cemented, accompanied by post-cementation instructions.

**Figure 2:**
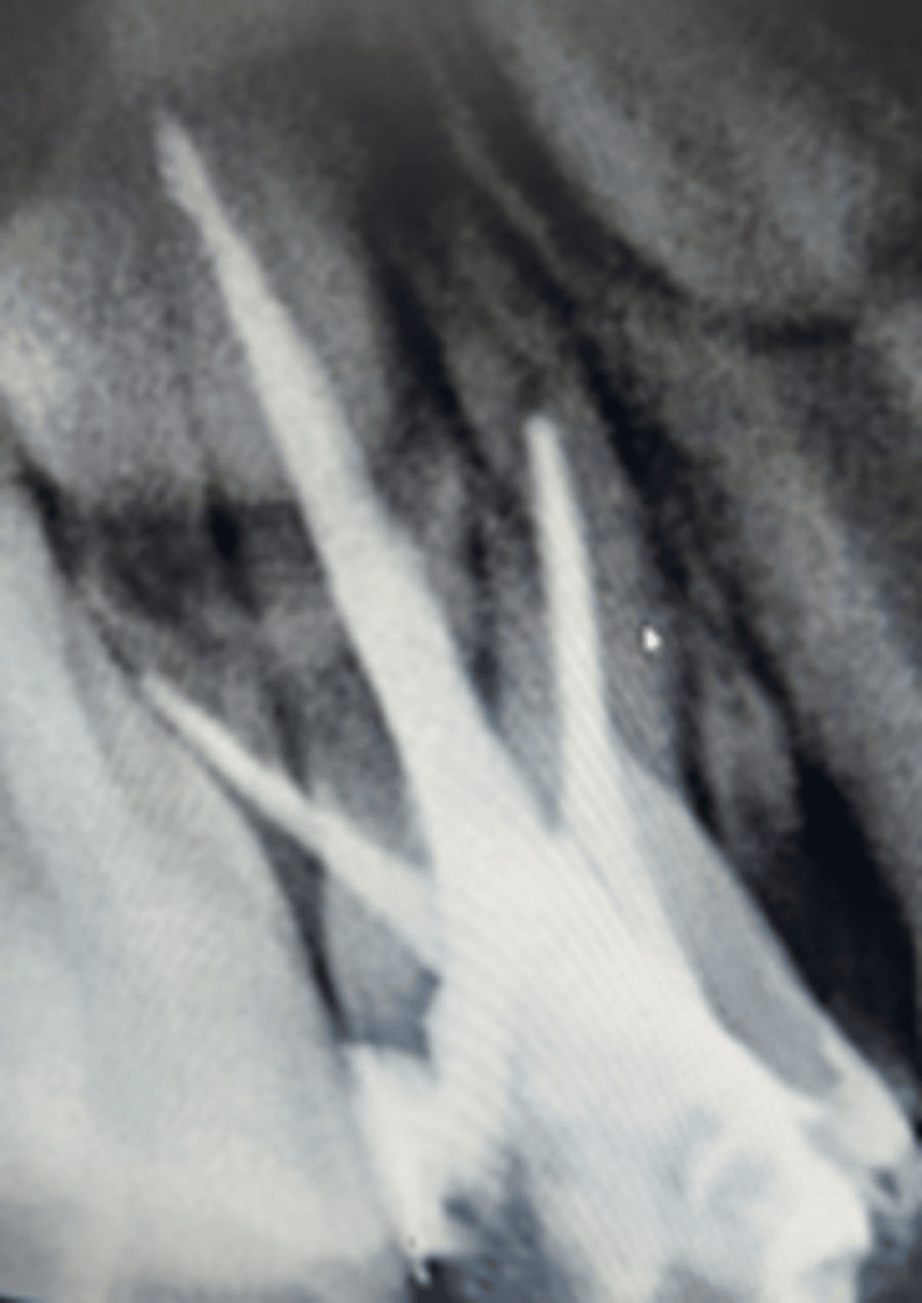
Post-obturation radiograph with respect to 54

**Figure 3:**
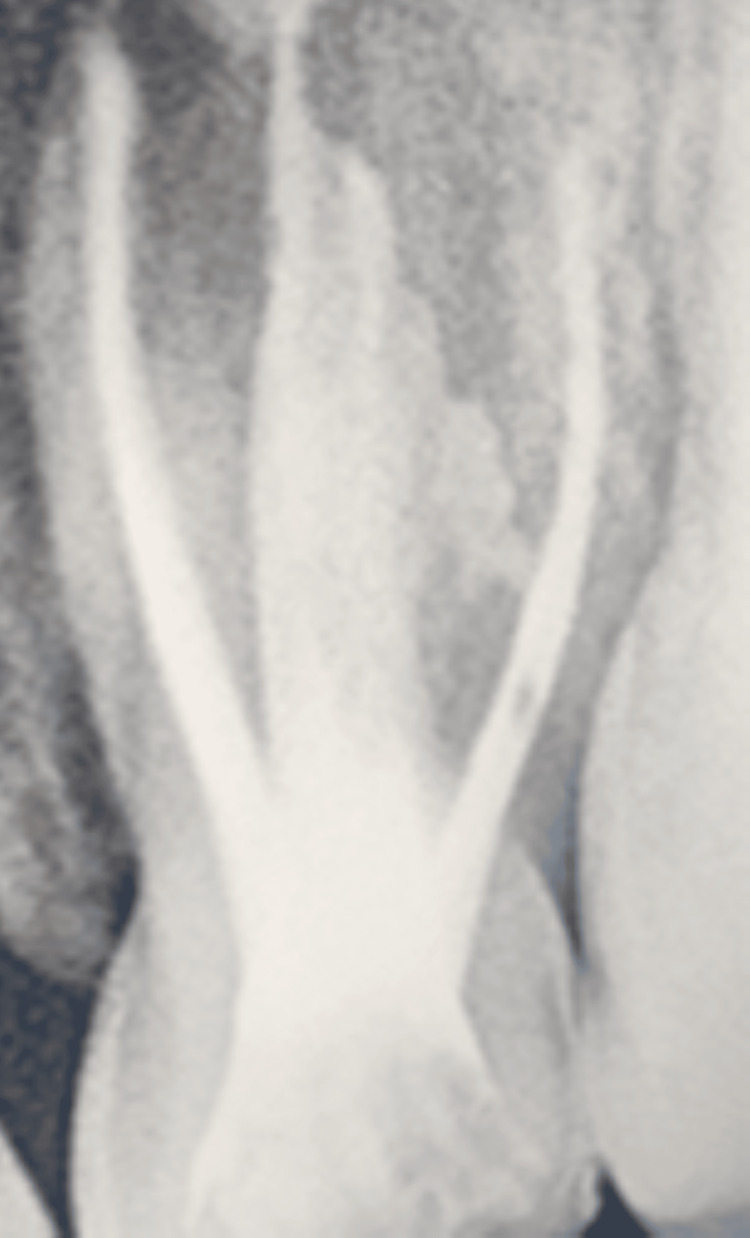
Post-obturation radiograph with respect to 64

**Figure 4:**
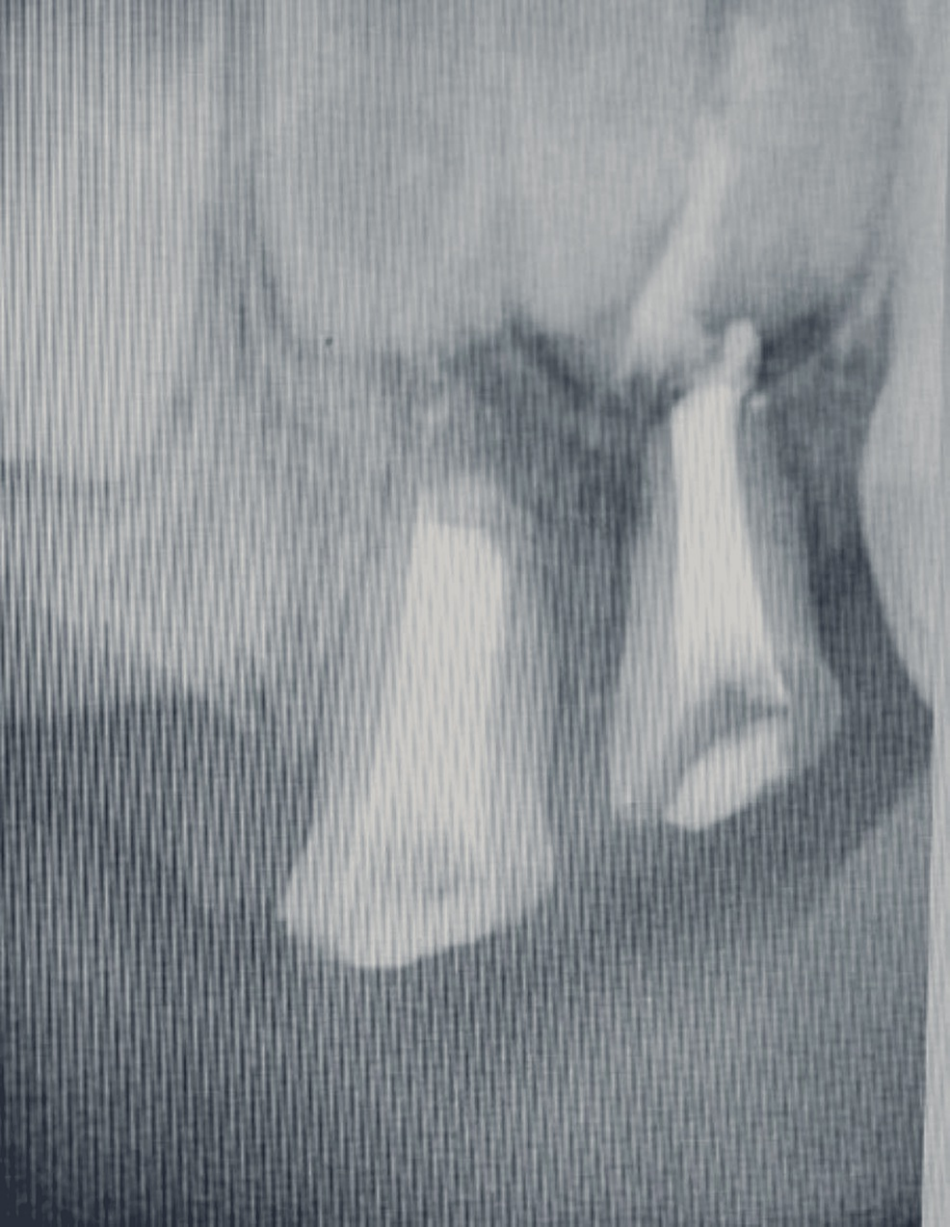
Post re-obturation radiograph with respect to 61 and 62

In the subsequent dental appointments, employing the tell-show-do technique, voice control, distraction, and positive reinforcement proved effective in enhancing the patient's cooperation with dental treatment. As per the treatment plan, initially, pulpectomy was conducted under local anesthesia (2% lidocaine with 1:80,000 epinephrine (Xicaine; ICPA Health Products Ltd., Mumbai, India), involving obturation of the canals with metapex (DiaPex® Plus; DiaDent Group International, Cheongju-si, Korea) and sealing with glass ionomer cement (XtraCem, Shade A2; Medicept UK Ltd., Harrow, UK) for teeth 54 (Figure 2) and 64 (Figure 3) as visible in the images. Following this, a re-pulpectomy was performed under local anesthesia for teeth 61 and 62, involving the obturation of canals with metapex (Figure 4).

Re-pulpectomy was done as a preventive measure to eliminate the risk of reinfection, given the absence of final restoration for a duration of three months.

**Figure 5:**
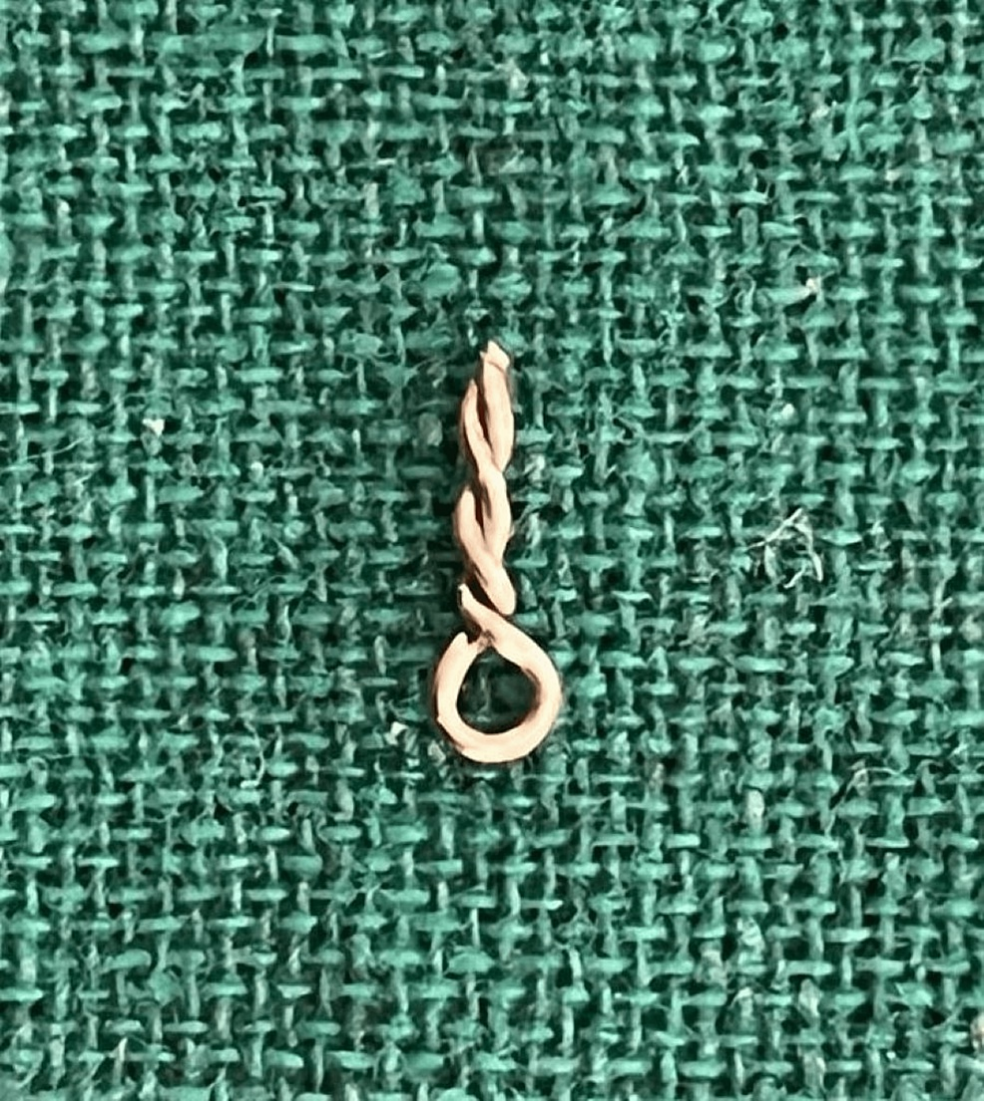
Modified omega loop in a twisted spiral design

Additionally, custom-made modified omega loops in a twisted spiral design were crafted from 22 gauge/0.7 mm stainless steel orthodontic wire (Round Smith Stainless Steel Wire, K.C.Smith & Co.) (Figure 5).

**Figure 6:**
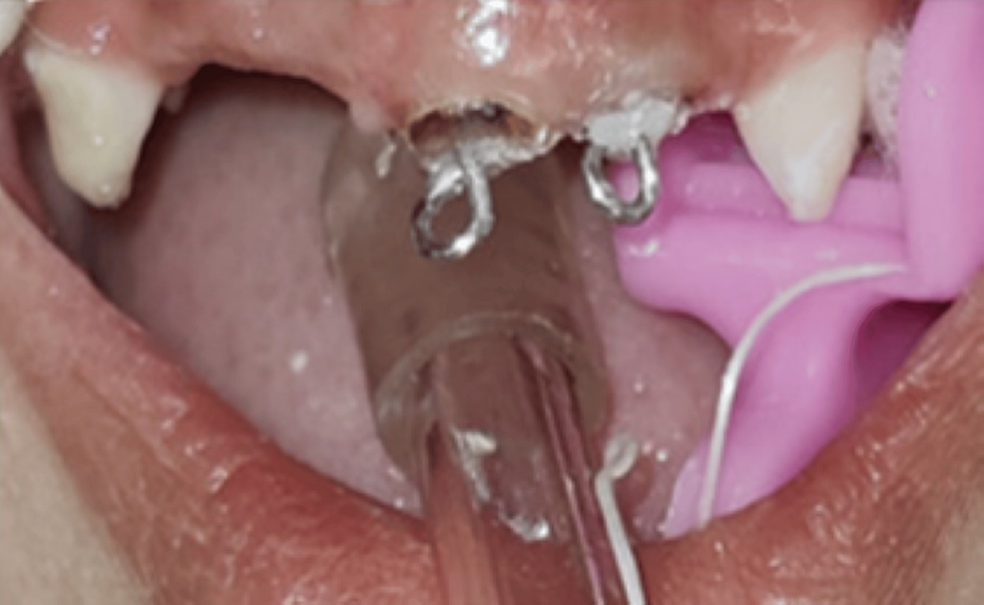
Clinical photograph of posts inserted and luted with 61 and 62, respectively

Twists were integrated to improve the stability of the aesthetic restoration and enhance the mechanical retention of the core. The posts were luted in the prepared root canals 61 and 62 using glass ionomer luting cement (XtraLute, Medicept UK Ltd., Harrow, UK) (Figure 6).

**Figure 7:**
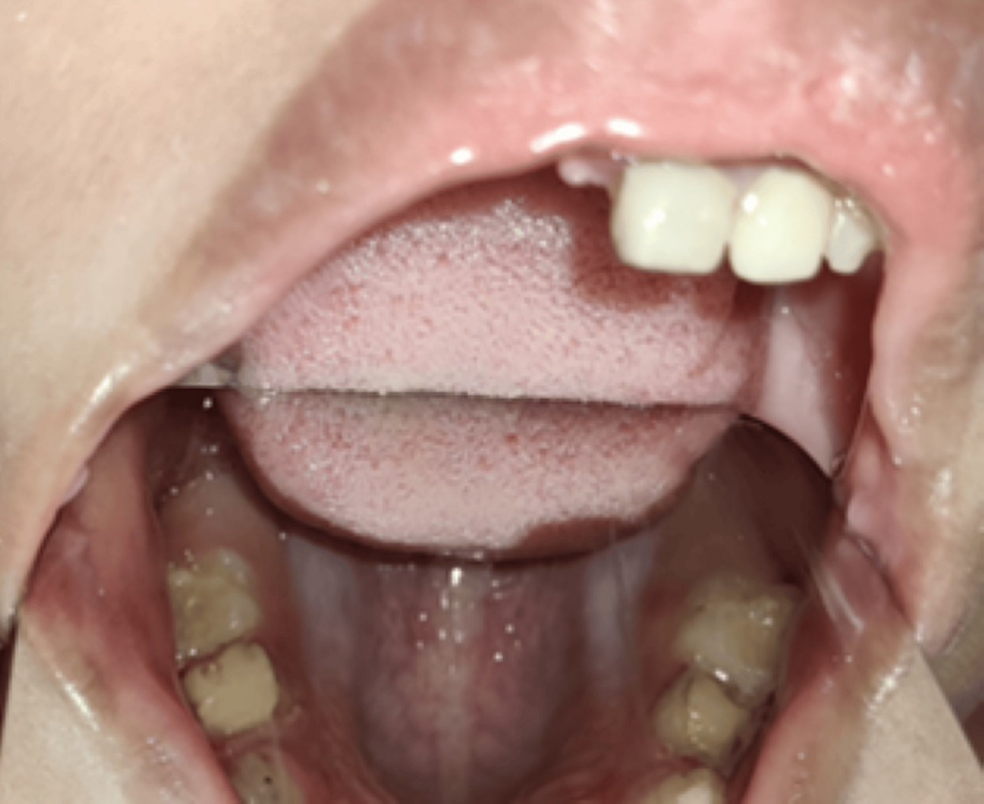
Clinical photograph showing coronal build-up with respect to 61 and 62

Subsequently, a composite (Therafil-31 Dental Composite Kit; Latus, Kharkov, Ukraine) of shade A2 was used to build up the coronal part in incremental technique (Figure 7).

**Figure 8:**
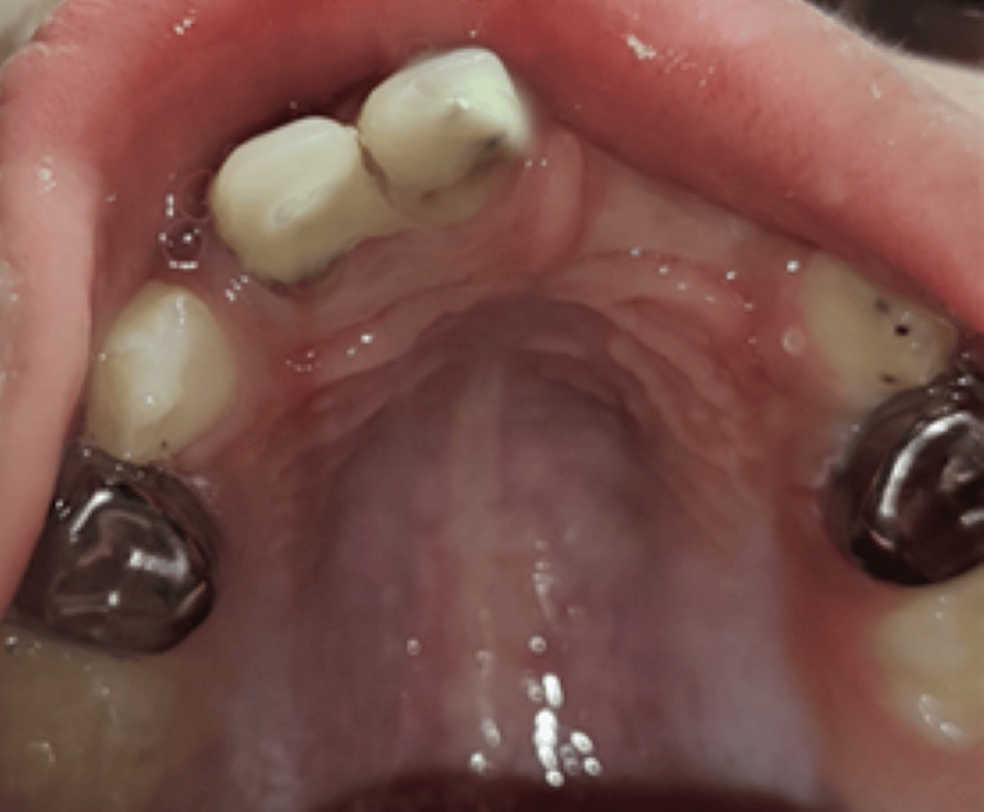
Maxillary view achieved using intraoral mirror demonstrating stainless steel crown adaptation with respect to 54 and 64 after endodontic procedures

Glass ionomer cement restorations were performed with 74 and 84, subsequently followed by the application of Fluoride in both arches. Following this, the stainless steel crown (Oro SS Little Crown Primary Molar Kit, Reach Global India Pvt Ltd., Pune, India) was adapted by proper contouring and crimping to achieve an optimal marginal fit for teeth 54 and 64, without cementing it to the teeth (Figure 8).

**Figure 9:**
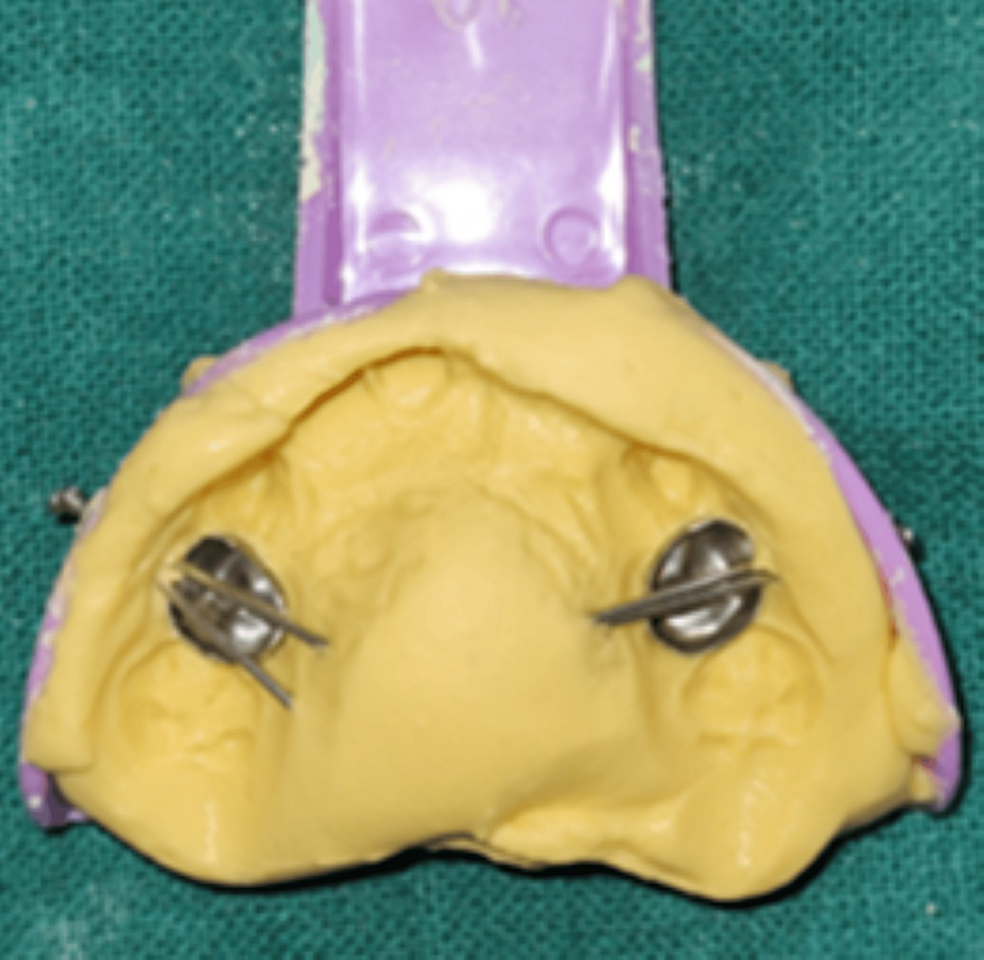
Alginate impression with the stainless steel crowns (in relation to 54 and 64) stabilized using the pin method

After placing the stainless steel crown (SSC) on the teeth, an alginate impression was made, and the SSCs were transferred onto the impression, securing their position using the pin method (Figure 9).

Subsequently, casts were created by pouring dental stones.

Fabrication of the modified Groper's appliance

**Figure 10:**
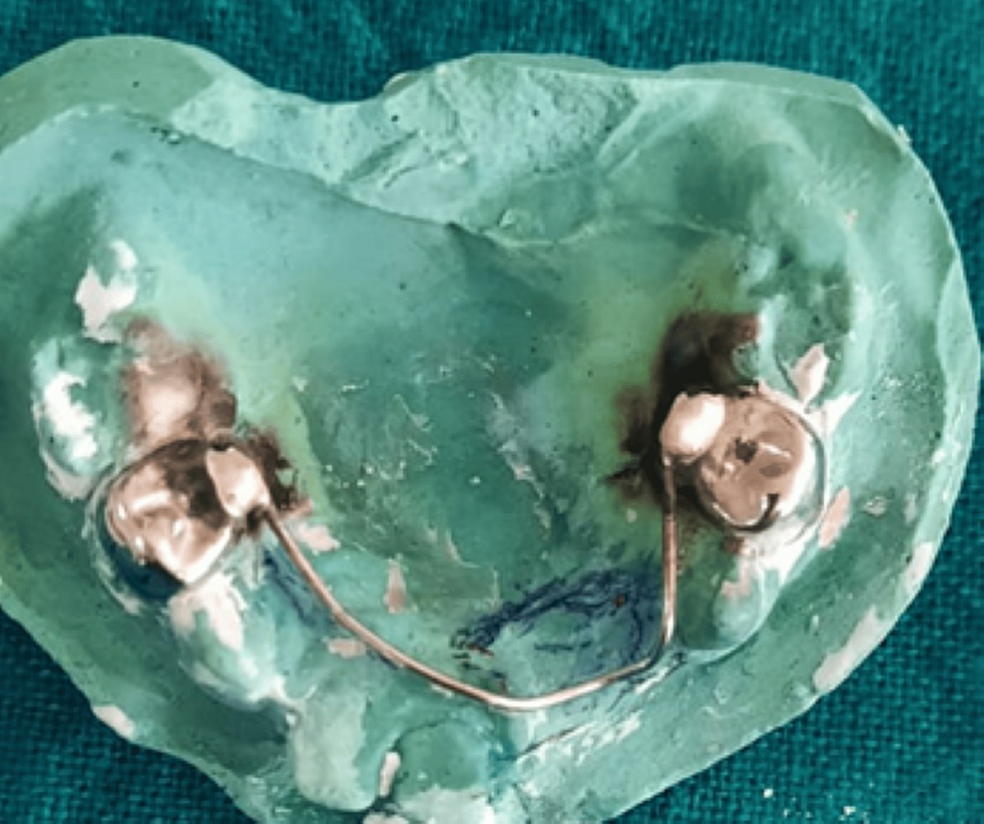
Working model with the initial wire framework soldered to the stainless steel crowns adapted to 54 and 64

One study model and a working model were prepared. The initial wire component, mirroring the contour of the maxillary arch, was crafted using a 21-gauge/0.8 mm diameter stainless steel orthodontic wire. This configuration extended from the palatal surface of the SSC on the first primary molar (54) to the palatal surface of the SSC on the corresponding first primary molar (64). Once the wire component was stabilized, the wire was soldered to the crowns (Figure 10).

**Figure 11:**
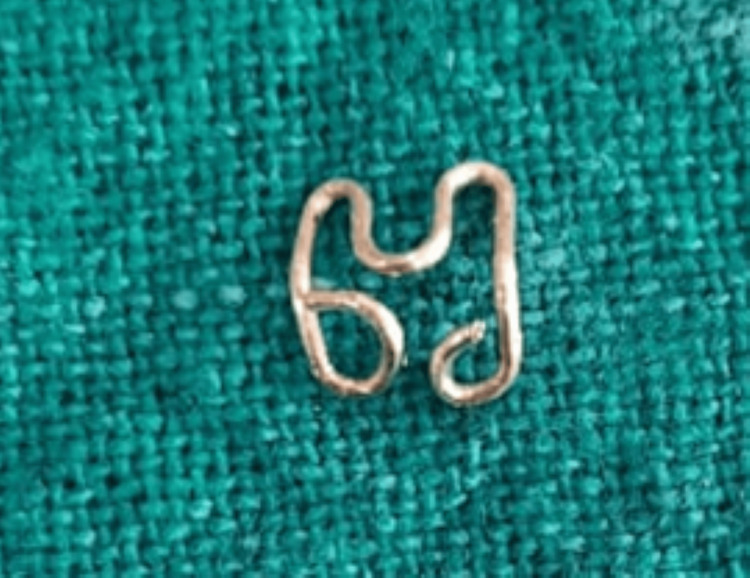
An additional crib-like wire component for the edentulous maxillary region (51 and 52)

The second wire component was created for the aesthetically concerned edentulous area of the maxillary arch, utilizing a 21-gauge/0.8 mm stainless steel orthodontic wire (Figure 11).

**Figure 12:**
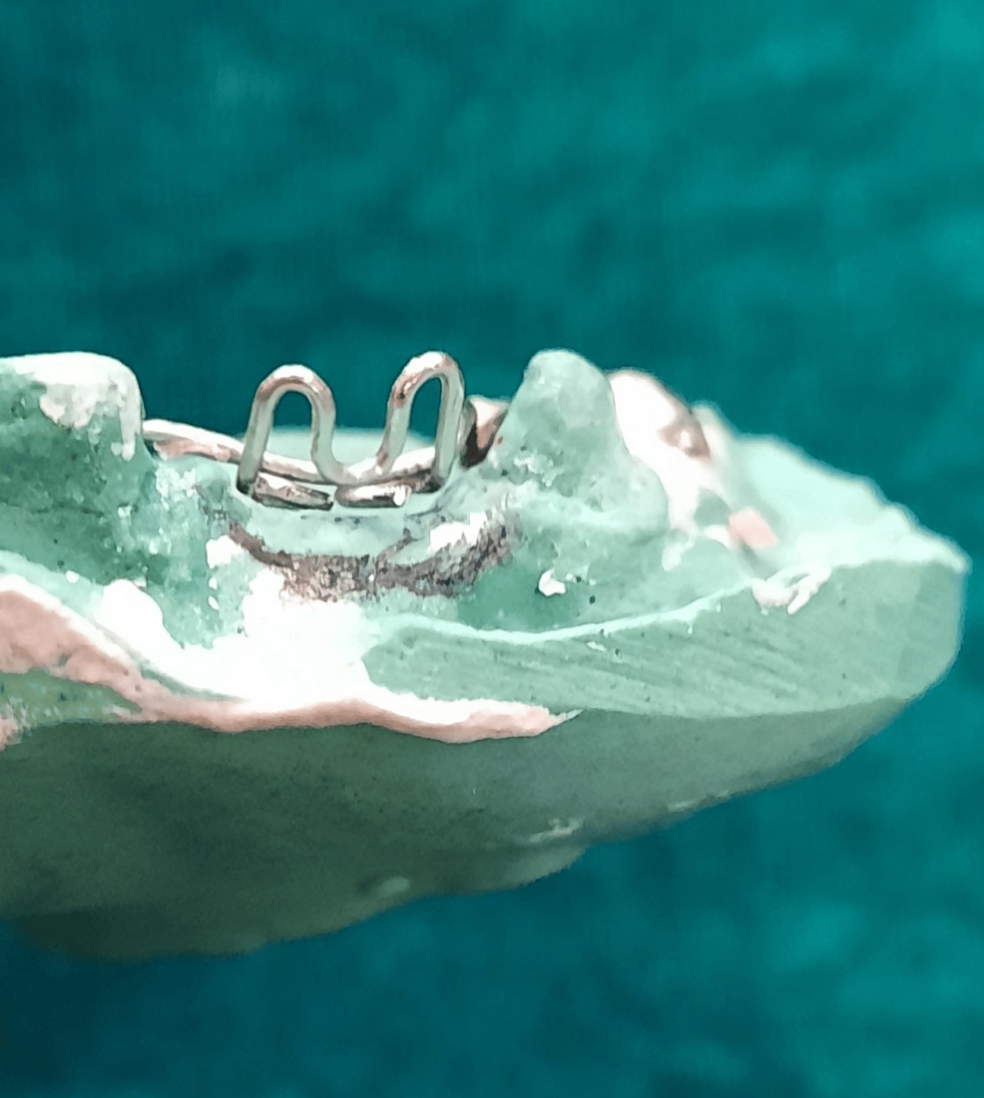
Each loop representing a missing tooth to be replaced in the crib-like wire component design

Using a universal plier (GDC UNIVERSAL PLIER (3000/55); GDC Fine Crafted Dental Pvt. Ltd., Hoshiarpur, India), it was bent in a crib-like fashion where the composite will lock itself during coronal part buildup (similar to the omega post). The number of loops in the wire corresponded to the number of teeth to be replaced, two in this case (51 and 52) (Figure 12).

**Figure 13:**
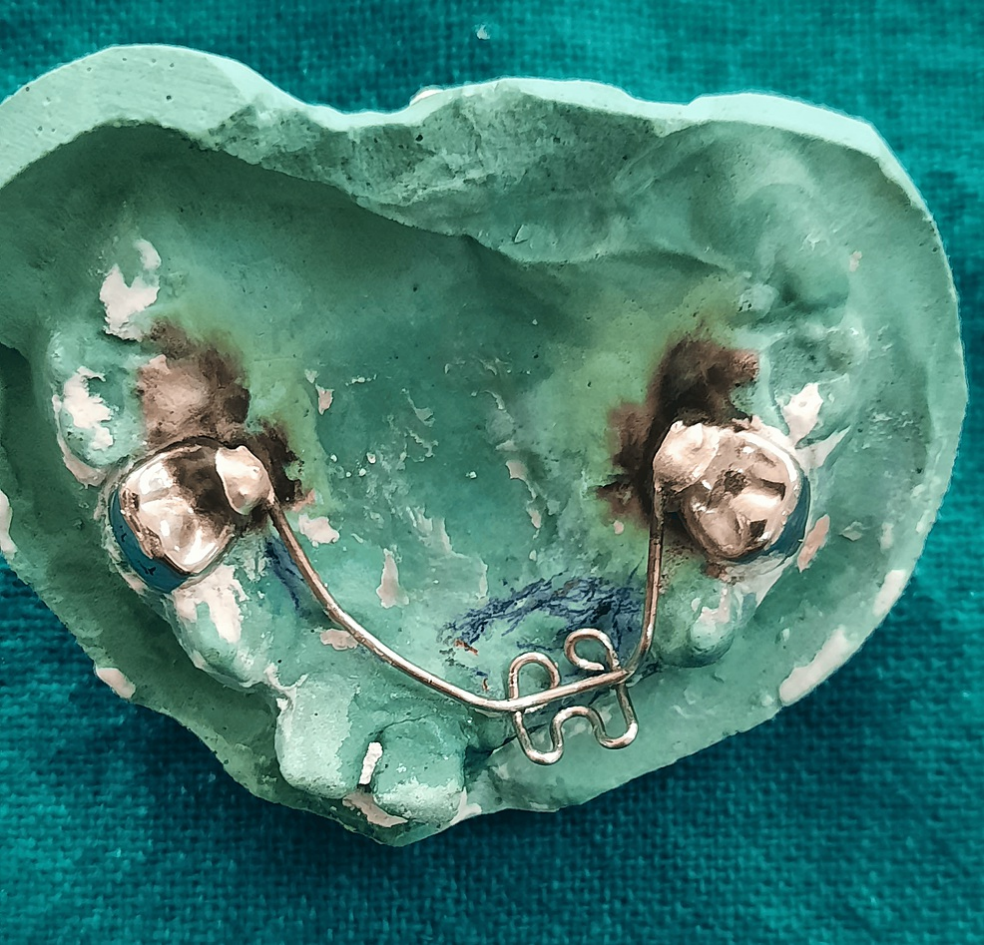
Retentive tags on the palatal aspect of the additional wire component

Retentive tags were made on the palatal aspect of the second wire component, intended to be embedded in acrylic subsequently, ensuring retention (Figure 13).

**Figure 14:**
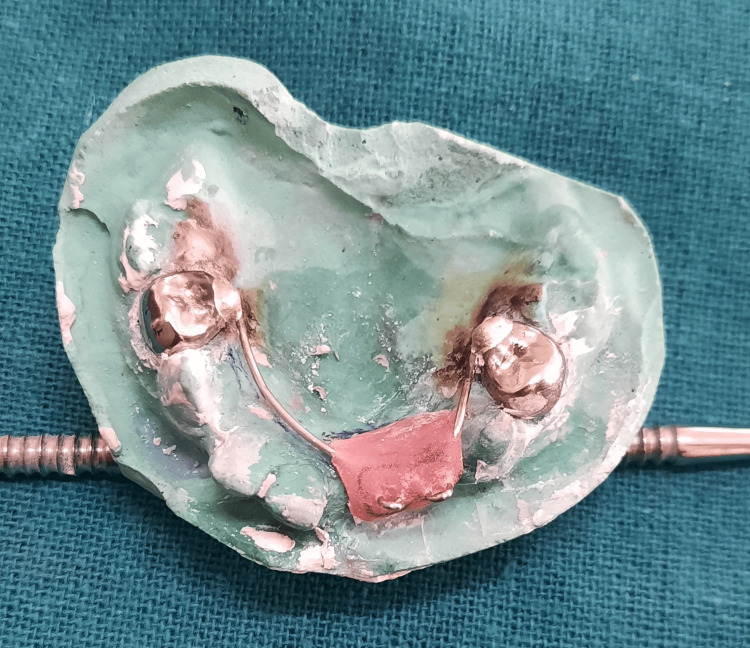
Maxillary cast with additional wire component acrylized in the edentulous area

**Figure 15:**
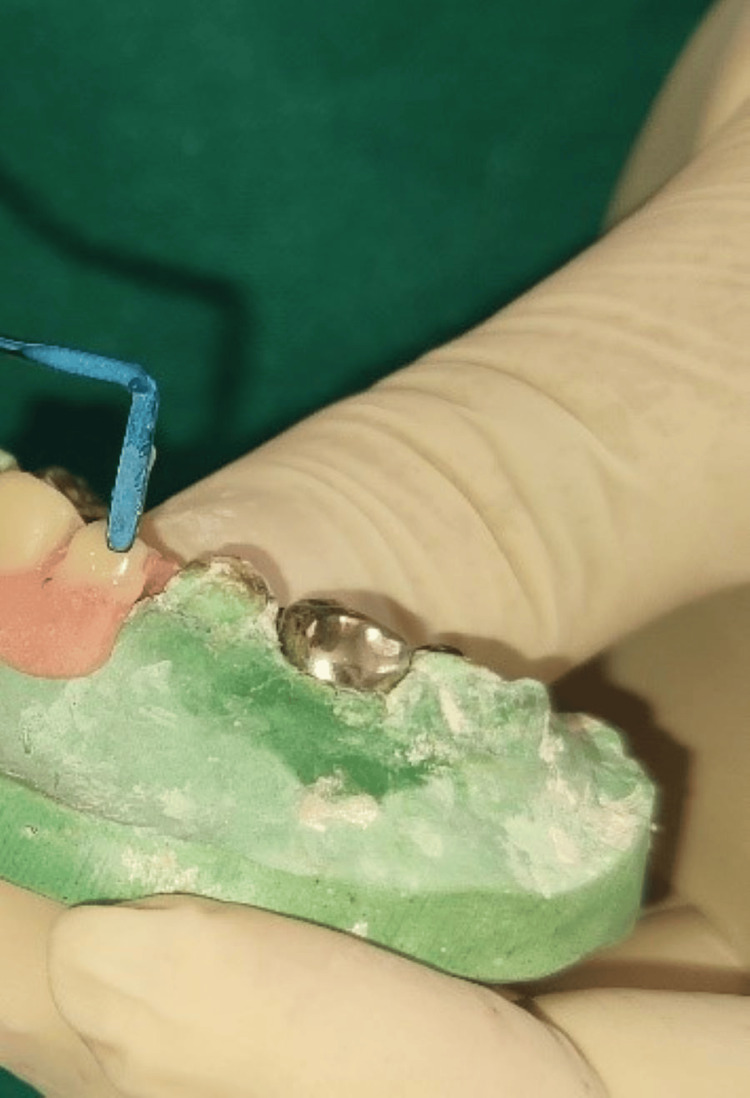
Crown build-up using the same shade of composite over the acrylized additional wire component

**Figure 16:**
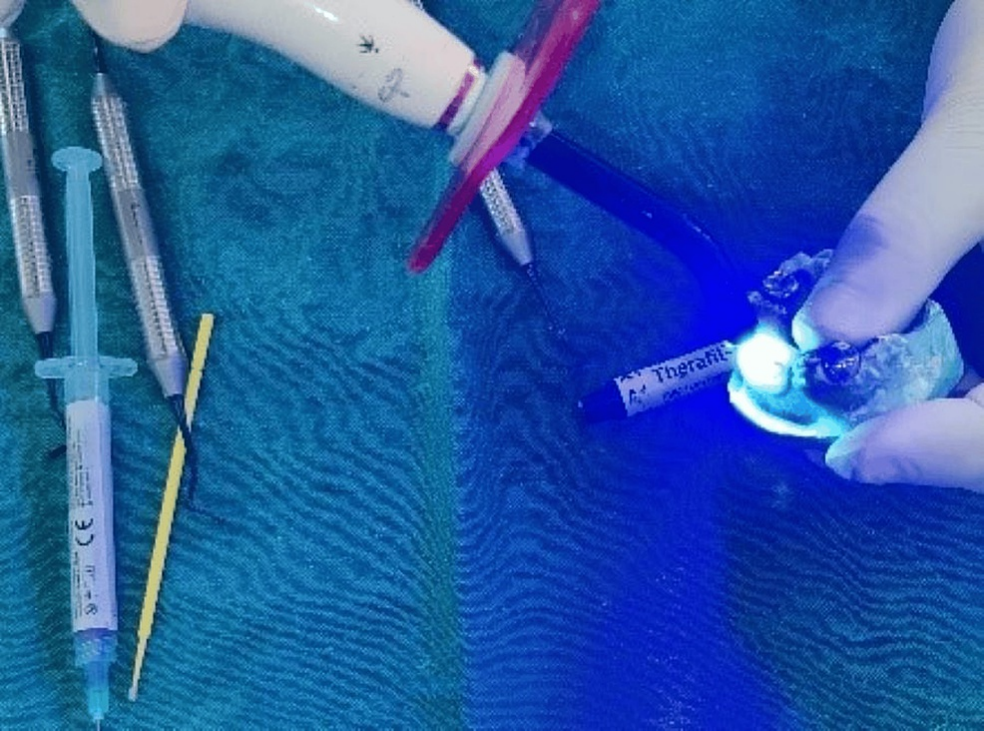
The crown build-up being performed directly on the cast

Subsequently, the second wire component was acrylized (Figure 14) and the crown buildup using the same shade of composite (Figure 15) was accomplished directly on the cast to replace the edentulous region in relation to 51 and 52 (Figure 16).

**Figure 17:**
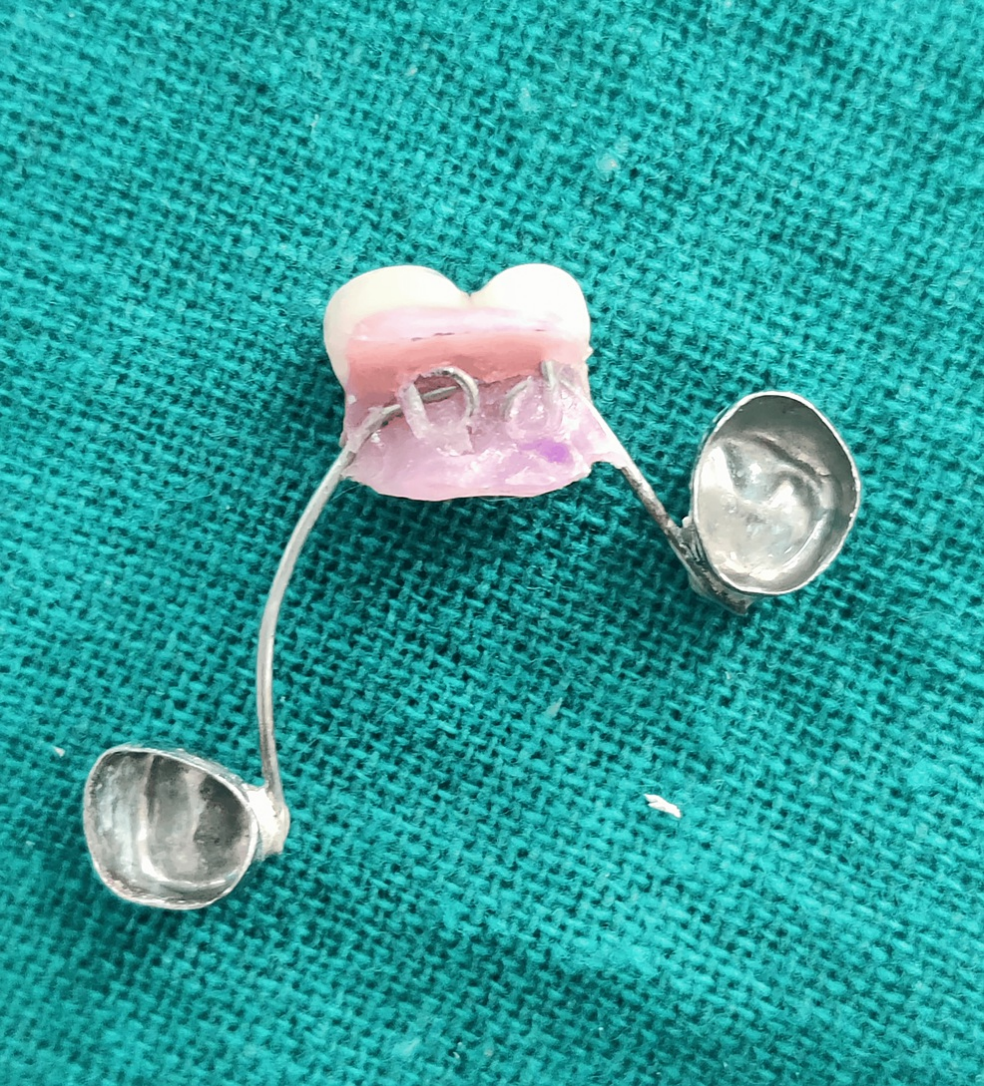
Composite interlocking within the loop of the additional wire component during the coronal portion buildup

The composite will interlock in the loop during coronal part build-up (Figure 17).

**Figure 18:**
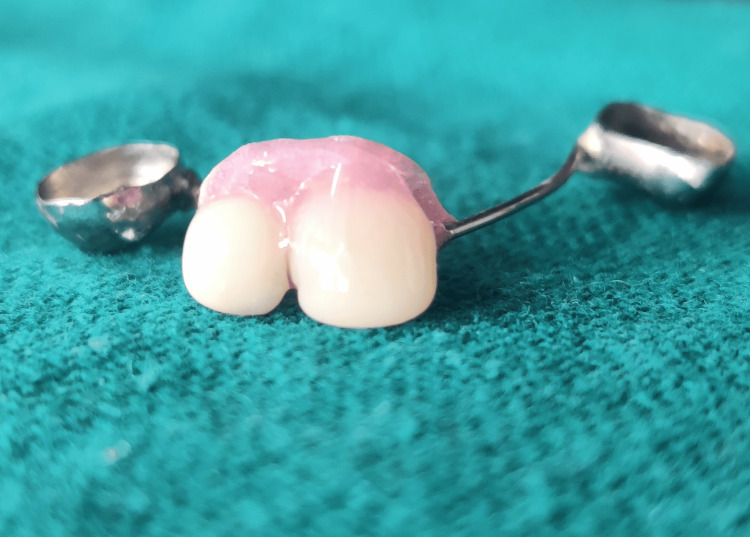
Trimmed, finished, and polished appliance

Etching was done to clean and roughen the loop wire surface. All essential trimming and polishing were then performed (Figure 18).

**Figure 19:**
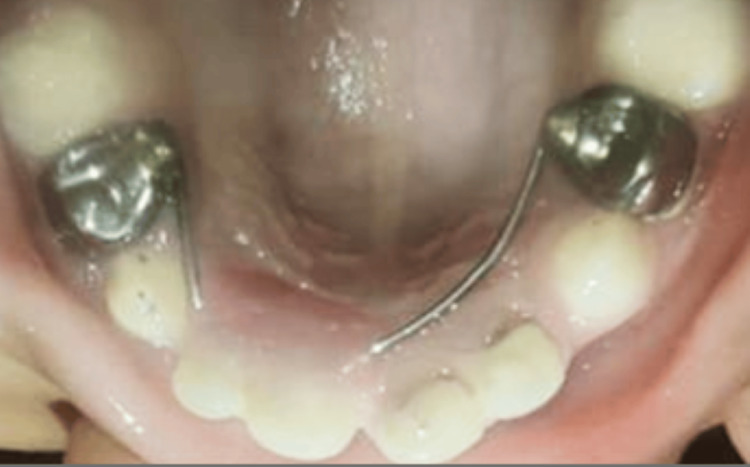
Maxillary view achieved using intraoral mirror showing cementation of the appliance after luting stainless steel crowns

The appliance was carefully cemented with glass ionomer luting cement, and a thorough occlusion check was conducted (Figure 19).

**Figure 20:**
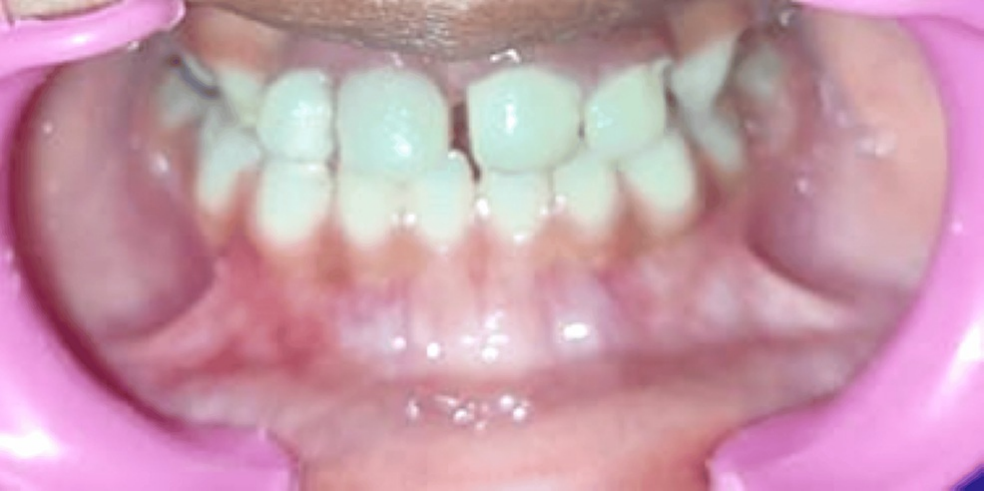
Post-cementation frontal view after 24 hours

Dietary and oral hygiene instructions were given to the parents and the patient. Follow-up examination after 24 hours was done, and a recall visit after every two months was advised (Figure 20).

The child and parents expressed contentment with the restoration of the lost teeth. The parents were briefed that the appliance would be removed approximately between the ages of 5.5 and six years to avoid hindrance to erupting permanent successors. The patient received guidance to steer clear of consuming hard or sticky and sugary foods and was recommended to uphold optimal oral hygiene practices. Additionally, the child was instructed to promptly return if any issues with the appliance, such as distortion or breakage, arose.

**Figure 21:**
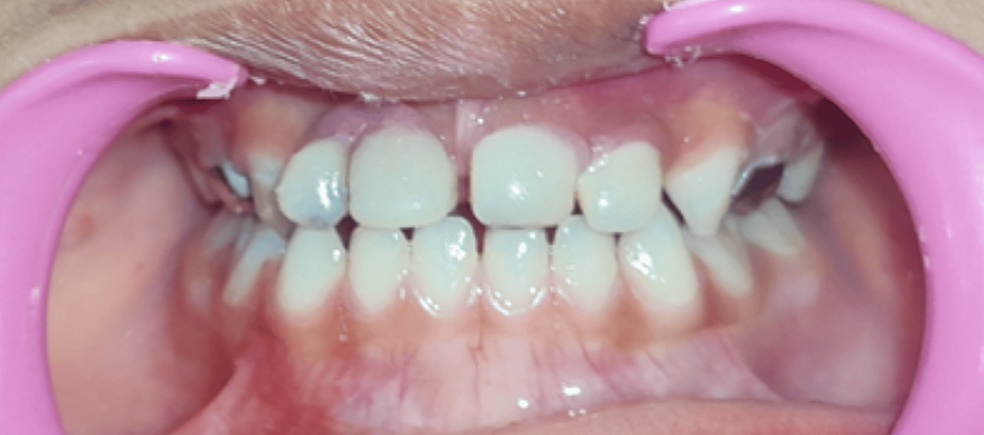
Frontal view after six months follow-up

During the subsequent follow-up appointments (Figure 21), it was observed that the boy appeared well-nourished and joyful. The child's mother reported an enhancement in the boy's overall lifestyle and behavior. The oral hygiene of the patient was deemed satisfactory, and the importance of maintaining it at the same level was conveyed.

## Discussion

In children below the age of three, the presence of any smooth-surface caries signifies S-ECC. Between ages three and five, the diagnosis of S-ECC includes one or more cavitated, missing teeth (because of caries), or filled smooth surfaces in primary maxillary anterior teeth. Additionally, a decayed, missing, or filled score of ≥4 (at age three), ≥5 (at age 4), or ≥6 (at age five) surfaces is indicative of S-ECC [6]. Consequently, our patient received a diagnosis of severe early childhood caries (S-ECC).

The untimely shedding of primary teeth may lead to orthodontic issues such as crowding, ectopic eruption, or tooth impaction, potentially resulting in malocclusion [7]. A study was conducted revealing that the absence of primary anterior teeth before the age of three years is correlated with speech difficulties [8]. There is an impact on the child's speech development after the removal of primary incisors, leading to distortion. If these teeth are absent, there is a potential for the development of inappropriate speech as the labiolingual sounds involve the tongue making contact with the lingual side of the maxillary incisors [5]. Therefore, providing this appliance was crucial for preserving the patient’s speech efficiency.

Premature tooth loss, particularly in the anterior region, can also give rise to psychosocial challenges for children [7]. The primary challenge in our field is to restore these patients aesthetically and functionally, addressing the psychological impact on both the patients and their parents. The desire expressed by parents stands out as a crucial determining factor in approaching these specific clinical situations [8]. The most appropriate space maintainer for a pediatric patient is the fixed type, as they are readily accepted [9]. Along with children having seizure disorders, immunocompromised status, mental retardation, substantial deep-bite, overjet, or anterior crossbite issues, the contraindications for placement of anterior fixed appliances also include challenges in follow-up, inadequate hygiene, and inappropriate feeding habits [10]. Therefore, following a thorough evaluation of the parent's interest in both the aesthetics and dental health of the child, the implementation of this appliance was carefully planned.

The utilization of fixed prostheses in children may be constrained by changes in dental arches during the progression from primary to mixed dentition occlusions. However, there exists a stable phase, typically between three and 5.5 years of age, during which a fixed appliance can be given. This period corresponds to the completion of the primary arch and the preservation of unaltered sagittal and transverse dimensions [11]. In this case, the appliance fabrication was performed on a patient approaching the stable phase; thus, it was a suitable and steady timeframe for such an intervention.

The initial Groper's appliance, as introduced by Jasmin and Groper in 1984, featured acrylic teeth affixed to metal clefts that were soldered onto the palatal wire. Recognizing the challenges posed by complex laboratory procedures, the appliance underwent modification, incorporating preformed acrylic teeth and a buccal acrylic flange [12].

Now, the introduced extra wire component within our modified Groper’s appliance prompts various benefits and notably improved aesthetics through composite shade matching, streamlining the crown buildup process. This proves especially advantageous in remote areas where acquiring a set of deciduous acrylic teeth may pose challenges. Notably, it eliminates the requirement to trim permanent acrylic teeth for a deciduous appearance and circumvents the associated time and cost of the laboratory fabrication of heat-cure acrylic crowns. This appliance provides numerous benefits, including restoration of masticatory and speech efficiency, improvements in aesthetics, and prevention of abnormal oral habit development.

However, similar to a well-balanced coin, this appliance exhibits a duality, featuring both commendable advantages and notable disadvantages. Such fixed appliances encompass the need for complete removal when the first upper permanent central incisor emerges. Another drawback involves the gathering of food debris and plaque. Therefore, parents should be advised to oversee their child's oral hygiene diligently and to show up at scheduled follow-up examinations [5,12].

## Conclusions

Timely intervention can mitigate the psychosocial challenges associated with the premature loss of primary teeth. The restoration of aesthetics and function using this appliance provided a substantial psychological uplift for the child. This distinctive case report underscores the creation of a modified Groper's appliance for the dental rehabilitation of a child dealing with ECC. The appliance received positive acceptance, and no unfavorable outcomes were documented during the follow-up sessions.
